# Supplementary material for: Developmental Vitamin D Deficiency in the Rat Impairs Recognition Memory, but Has No Effect on Social Approach or Hedonia
Source: Nutrients. 2019 Nov 8;11(11):2713. doi: 10.3390/nu11112713 (PMC6893501; doi:10.3390/nu11112713)
Supplement: Supplementary file 1 [file nutrients-11-02713-s001.zip › supplementary/Figure S1 Novel Objects.pdf]

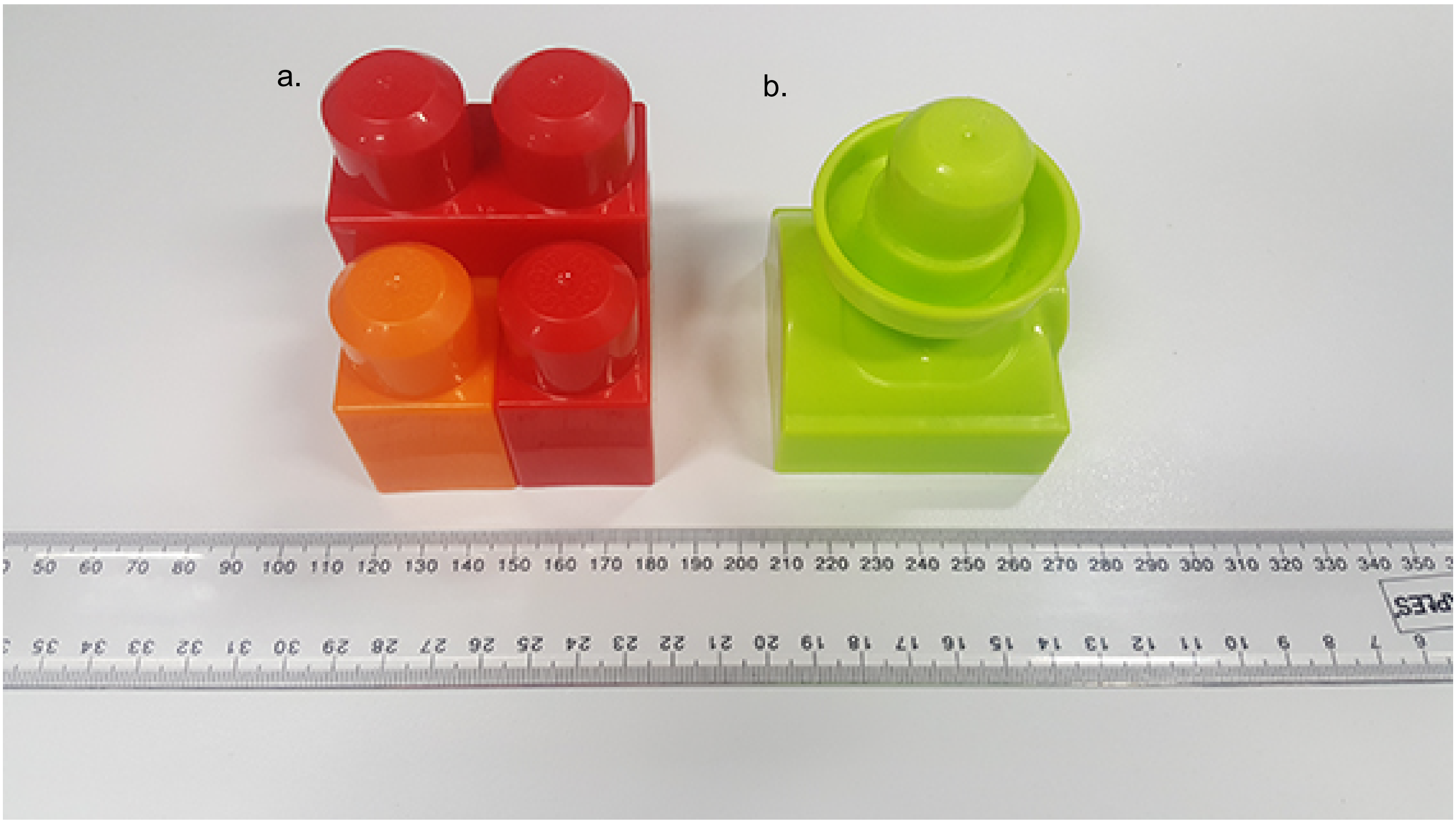

Figure S1. Objects used in the NORT test. a. The familiar object (NB: two of these objects were used for familiarisation). b) The novel object.
