# Supplementary figures and images for: Developmental Vitamin D Deficiency in the Rat Impairs Recognition Memory, but Has No Effect on Social Approach or Hedonia
Source: Nutrients. 2019 Nov 8;11(11):2713. doi: 10.3390/nu11112713 (PMC6893501; doi:10.3390/nu11112713)

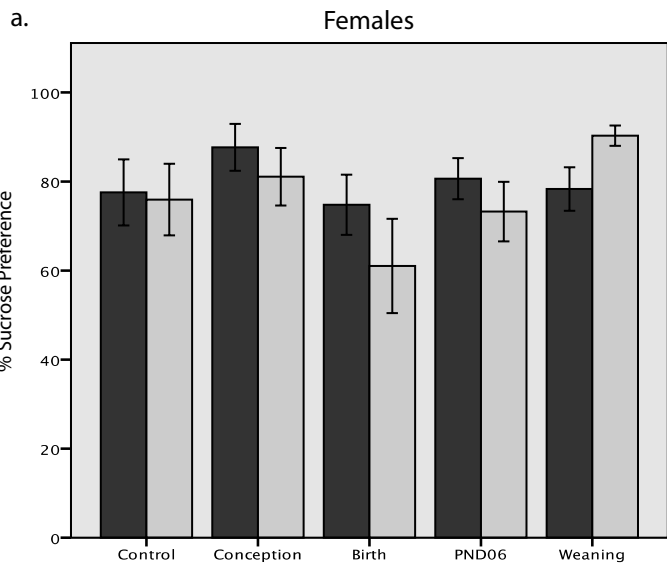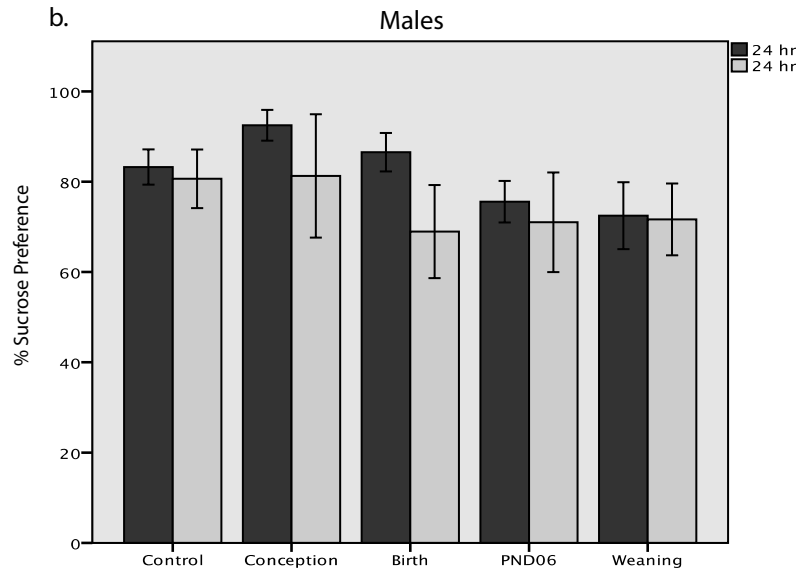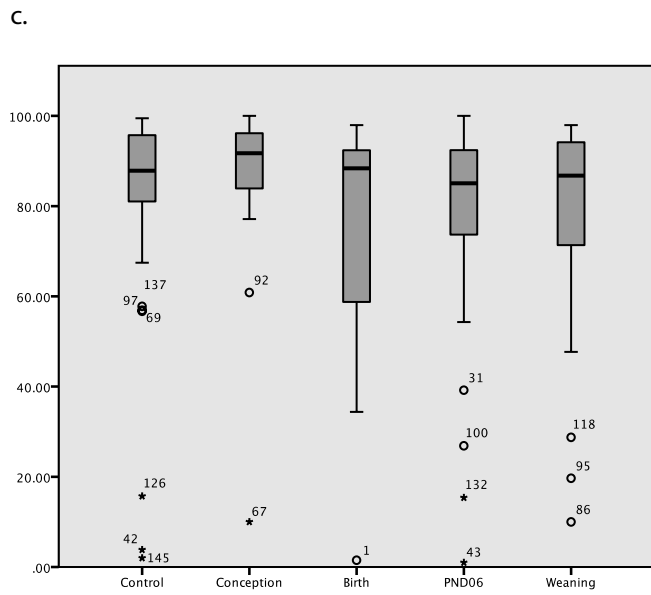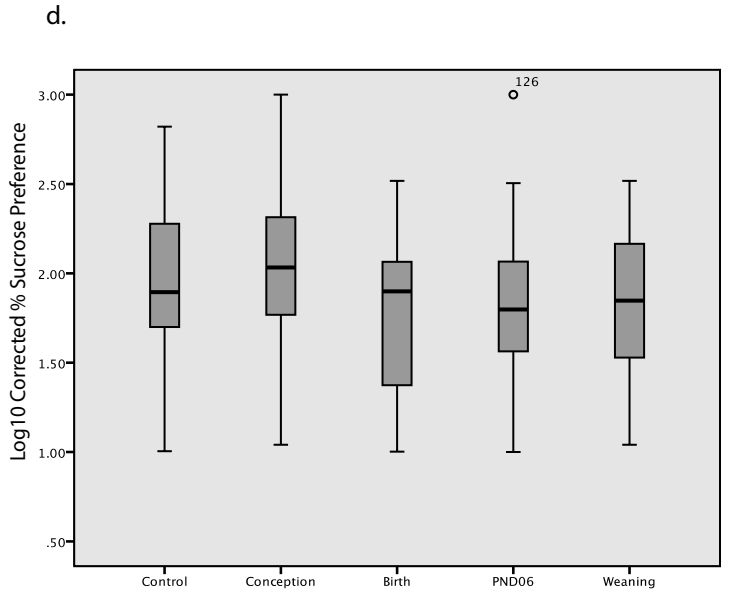

Supplement: Supplementary file 1 [file nutrients-11-02713-s001.zip › supplementary/Figure S2 Normalisation of Sucrose Pref.pdf]
